# Severe Respiratory Syncytial Virus Bronchiolitis in Infants Is Associated with Reduced Airway Interferon Gamma and Substance P

**DOI:** 10.1371/journal.pone.0001038

**Published:** 2007-10-17

**Authors:** Malcolm G. Semple, Hinke M. Dankert, Bahram Ebrahimi, Jailson B. Correia, J. Angela Booth, James P. Stewart, Rosalind L. Smyth, C. Anthony Hart

**Affiliations:** 1 Division of Child Health, School of Reproductive & Developmental Medicine, University of Liverpool, Liverpool, United Kingdom; 2 Division of Medical Microbiology and Genito-Urinary Medicine, School of Infection and Host Defence, University of Liverpool, Liverpool, United Kingdom; Duke University, United States of America

## Abstract

**Background:**

Severe human respiratory syncytial virus (hRSV) bronchiolitis in previously well infants may be due to differences in the innate immune response to hRSV infection. Aim: to determine if factors mediating proposed mechanisms for severe bronchiolitis differ with severity of disease.

**Methodology/Principle Findings:**

197 infants admitted to hospital with hRSV bronchiolitis were recruited and grouped according to no oxygen requirement (n = 27), oxygen dependence (n = 114) or mechanical ventilation (n = 56). We collected clinical data, nasopharyngeal aspirate (NPA) and if ventilated bronchoalveolar lavage (BAL). Interferon-gamma (IFN-γ), substance P (SP), interleukin 9 (IL-9), urea and hRSV load, were measured in cell free supernatant from NPA and BAL. Multivariate analysis compared independent effects of clinical, virological and immunological variables upon disease severity. IFN-γ and SP concentrations were lower in NPA from infants who required oxygen or mechanical ventilation. Viral load and IL-9 concentrations were high but did not vary with severity of disease. Independent predictors of severe disease (in diminishing size of effect) were low weight on admission, low gestation at birth, low NPA IFN-γ and NPA SP. Nasal airway sampling appears to be a useful surrogate for distal airway sampling since concentrations of IFN-γ, SP, IL-9 and viral load in NPA correlate with the same in BAL.

**Conclusions:**

Our data support two proposed mechanisms for severe hRSV disease; reduced local IFN-γ response and SP mediated inflammation. We found large amounts of hRSV and IL-9 in airways secretions from the upper and lower respiratory tract but could not associate these with disease severity.

## Introduction

Human respiratory syncytial virus (hRSV) infection is ubiquitous in infancy and it is the single most important cause of severe lower respiratory tract infection in young children throughout the world.[1] Severe disease (bronchiolitis or bronchiolitis pneumonia) results in the hospital admission of approximately 2.5% of all infants in industrialised countries making this virus the most common cause for admission in the first year of life.[2] In developing countries the morbidity and mortality associated with hRSV infection is far greater.[3] Approximately 50% of infants admitted to hospital with bronchiolitis suffer from recurrent wheeze in later childhood.[4], [5] It is unknown whether the mechanism(s) associating severe bronchiolitis with recurrent wheeze are a consequence of hRSV infection or due to an inherent susceptibilities to both conditions.

The histopathology of severe bronchiolitis is small airway obstruction due to viral-cytopathic epithelial disruption and destruction, parenchymal inflammatory-cell infiltration, luminal neutrophil migration and increased mucous production.[6], [7] Mechanisms recently proposed to explain severe bronchiolitis include reduced antiviral immunity (reduced IFN-γ production), increased viral replication, excessive “neurogenic inflammation” and elevated IL-9-induced secretion of mucous.[8]–[11] These mechanisms are based upon experimental studies in animals and observational studies in well defined but relatively small groups of human infants.

In this large pragmatic clinical observational study, each mechanism in this pathogenic pathway was tested by measuring representative factors (IFN-γ, viral load, Substance P and IL-9) in the respiratory secretions collected from infants admitted to hospital.

Our hypothesis is “where variation in a pathogenic mechanism is associated with severe disease, representative factors (cytokines and viral load) will vary with disease severity”.

We studied the interactions and independent effects of these factors upon disease severity by univariate and multivariate analysis in previously well infants. Where associations were found we compared the size of effect (relative odds ratio (ROR)) for each measured factor with the size of effect for known clinical and environmental risk factors for severe disease in the same groups of infants.

Previous studies by our group have shown significant differences in the disease characteristics and clinical course of hRSV bronchiolitis between other-wise healthy infants born at term and those born prematurely.[6], [10], [12] Because of these known differences and expected over-representation of premature infants with severe disease we included analysis of term and premature infants.

## Methods

### Participants

Infants admitted with bronchiolitis to Alder Hey Children's Hospital, Liverpool during the winter seasons (November through February) of 2002/3, 2003/4 and 2004/5 were recruited.

Bronchiolitis was diagnosed by paediatricians when infants (children <2 years old) presented with tachypnea (>50 breaths/min), subcostal recession, and bilateral inspiratory crackles on auscultation. hRSV status was established by a rapid antigen assay of nasopharyngeal aspirate (NPA) described below. Only hRSV antigen positive infants were included in this study. Criteria for admission to the general wards include reduced oral intake (<75% of normal) and/or oxygen saturation at <93% in air. Oxygen was administered to maintain oxygen saturation at >92%. Infants with respiratory failure or significant apnoea who required mechanical ventilation were admitted to the intensive care unit. Ventilated infants were intubated using an uncuffed naso-tracheal tube, nasal CPAP was not used.

Baseline data were collected at recruitment using a structured history-form which included queries regarding a family history of atopy in first degree relatives and current smoking by any household member regardless of situation (Appendix S1). Premature (pre-term) birth was defined as birth before completion of 37 weeks gestation. Infants who had previously been admitted to hospital or who had a history of haemodynamically significant congenital cardiac disease were excluded.

Infants were retrospectively grouped according to the worst severity of disease experienced during their admission using the following criteria: “never any need for supplemental oxygen”, “oxygen dependency at any time” and “any need for mechanical ventilation”.

### Collection of Nasopharyngeal Aspirate (NPA)

NPA was collected by nursing staff as part of the diagnostic process and for surveillance of community-acquired respiratory infections, usually during the process of admission (<3 hrs) and always within 24 hours of admission. Infants were swaddled in a small blanket and placed supine in a cot. Nasal secretions from both nostrils were aspirated without lavage using a soft size 8F catheter with two distal lateral eyes and a proximal side port for finger-tip suction control (CARETIP™ atraumatic-neonatal, Meddis Ltd UK) connected to a conical trap (Tracheal suction set, Unomedical Ltd UK) by medical vacuum (20 mmHg) while advancing and withdrawing the catheter tip to a depth of approximately 5 cm. The collection trap containing NPA was taken to a laboratory and refrigerated (+4°C) for further processing.

### Collection of bronchoalveolar lavage

Non-bronchoscopic bronchoalveolar lavage (BAL) samples were collected from ventilated infants by one of the investigators (MGS) or a experienced respiratory physiotherapist, in accordance with the European Respiratory Society 2000 guidelines and our earlier studies.[10], [13] A weight adjusted volume of 0.9% saline (1 ml/kg) was instilled twice and then for a minimal dwell time to minimise any urea dialysis effect.[14], [15] It is not possible to collect BAL from conscious non-intubated infants and we can not justify anaesthetising infants with bronchiolitis only to collect BAL for research purposes.

### Detection of hRSV

NPA sample was diluted with 3 ml of sterile 0.9% saline, mixed vigorously, and subjected to hRSV antigen enzyme linked immunosorbent assay (ELISA) (BD Directigen RSV™; Becton Dickinson UK). Residual NPA solution was refrigerated (+4°C) for further processing.

### Sample processing

BAL and residual NPA samples were filtered through nylon monofilament gauze with a 60 µm pore size (Sefar Nitex 03-48/31, Sefar Inc, Switzerland) to remove bulk mucus. The filtrate was centrifuged at 300 g for 5 minutes at +4°C, the supernatant removed, split into aliquots, and stored at −70°C.

### Measurement of Urea

A well defined denominator used in a homogenous population results in smaller coefficients of variation and a gain in precision of the measurement.[16] Respiratory sample urea concentration was used as a marker of sample quality and as denominator to adjust for respiratory sample dilution during collection and processing.[17] The urea dilution method for estimating dilution of respiratory samples has recently been validated in studies of cats and dogs.[18], [19] Urea was measured using a sensitive urease method with a minimum detection limit of 0.06 mmol/l (Randox Laboratories Ltd, UK).[20] Respiratory samples were excluded from further analysis where the urea concentration was less than 0.1 mmol/l, an arbitrary limit indicating excessive dilution.

Blood urea was only measured when there were concerns about possible dehydration. Routine venesection is specifically not part of the bronchiolitis care pathway of previously well infants at Alder Hey Children's Hospital and was not permitted by the ethics committee for this research project. Blood urea was measured in serum using a urease and glutamate dehydrogenase method (COBAS INTEGRA 700®, Roche Diagnostics). Blood urea values were collated when measured within 24 hr of admission.

### Measurement of cytokines in NPA and BAL

IFN-γ and SP were measured by ELISA (Diaclone, France and R&D Systems Europe Ltd, UK) as per manufacturer's instructions. The assay was validated for measurement of IFN-γ and SP in these respiratory samples by a spiking and recovery experiment involving addition and serial dilution of known amounts of IFN-γ and SP in a panel of 10 surplus samples (data not shown). Then further assays of IFN-γ and SP in a panel of 20 respiratory samples using increasing dilutions of assay diluent. The optimum dilution of respiratory samples for IFN-γ was 1 in 2 and for SP was 1 in 10. The minimum detection limit of IFN-γ was 5 pg/ml and for SP 8 pg/ml. IL-9 was measured using our validated in-house ELISA as described previously.[10] The minimum detection limit of IL-9 was 80 pg/ml. Cytokine concentrations in grouped data were compared “raw” and adjusted for dilution during sample collection and handling by the urea dilution method described above.

### Measurement of cell free hRSV load

Nucleic acid was extracted from 140 µL of filtered cell-free respiratory sample using QIAamp™ viral RNA MiniKit™ (Quiagen Ltd, UK). 10 µL of purified nucleic acid solution was subject to reverse transcription using a hRSV specific primer as previously described.[21] Two 1 µL samples of each cDNA solution were subject in duplicate to 35 cycles of real time polymerase chain reaction (RT-PCR) using primers specific to a highly conserved region of the hRSV N gene and an Opticon 2™ thermal cycler (Genetic Research Instrumentation Ltd., UK). Quantification was based on gene-specific standard curves. hRSV load is expressed as copies of N gene molecule per microlitre of sample (copies/µl). Details in Appendix S1.

### Statistics

*A priori* power calculations estimated that 16 participants were required in each group to observe a difference of 1 standard deviation (SD) (assuming 1 SD = 30 pg/ml) between group geometric mean cytokine concentrations with 80% power (α = 0.05 and β = 0.2). Statistical analyses were performed using the software SPSS v13.0.1 (SPSS Inc. Illinois, USA). Clinical characteristics of participants were compared by Pearson Chi-square test. Non-parametric cytokine data were compared by Mann-Whitney U test. Normally distributed cytokine and viral load data were compared by Student's t-test after validity was verified by Levene's test. Univariate statistical tests were two tailed and the p value is given when significant (p<0.05). Pearsons correlation coefficient and the measure of goodness of fit of the linear least-square model (R2) were calculated for group data in paired samples. Multivariate binary logistical regression models were constructed for two previously accepted definitions of severe disease “any need for supplemental oxygen including ventilation” and “any need for mechanical ventilation” [11], [22], [23]. Co-variables included *a priori* in the models were gender, birth-weight, gestation at birth, corrected age on admission, weight on admission, family history of atopy, exposure to environmental tobacco smoke, NPA concentrations of SP, IFN-γ, IL-9 and NPA hRSV load. History of apnoea was excluded from the model as it is a criterion for mechanical ventilation. Co-variables were rejected from the models where the effect was not significant (p>0.1). The assumptions of the model were tested by examination of the residuals and the overall fit of the model was ascertained using the Hosmer and Lemeshow goodness of fit test.[24]

### Ethics

The Liverpool Children's Local Research Ethics Committee, an independent statutory body, approved the study. Written informed consent was obtained from all parents or carers.

## Results

### Patient Characteristics

197 infants were recruited. Samples from 12 infants had urea concentrations below 0.1 mmol/l and were excluded from further analysis leaving 185 participants in the study. The distribution of excluded cases between disease groups was not significant.

There were significant differences between groups (table 1). There were significantly more prematurely born infants in the ventilated group than the other two groups (p<0.01). The ventilated group weighed less on admission, were younger (actual age and age corrected for gestation at birth) and were born earlier in gestation (all p<0.001). A significantly greater proportion of infants in the ventilated group presented with apnoea (P<0.01). Males were over-represented amongst all infants admitted with bronchiolitis but the proportions between disease severity groups did not differ significantly. There were no significant differences between the disease groups with respect to gender, family history of atopy, current smoking by any household member or duration of illness prior to admission. One premature infant in each disease group received intramuscular monoclonal anti-hRSV gamma-globulin prior to admission.

**Table 1:** Characteristics of participants.

Characteristic	Worst Disease Severity during admission		
	No oxygen n = 25	Oxygen n = 107	Ventilation n = 53
Male sex, n (%)	15, 60%	66, 62%	34, 64%
Premature birth (n)	7	19	24*
Term birth at (n)	18	88	29
Weight on admission(kg)	5.6 (4.9 to 7.3)	6.6 (4.8 to 8.2)	3.7 (3.0 to 4.8) **
Age on admission (weeks)	13 (7.9 to 21)	20 (11 to 32)	6.3 (4.2 to 13) **
Gestation (weeks)	40 (37 to 40)	40 (38 to 40)	37 (33 to 40) **
Corrected Age (weeks)	10 (6.1 to 18)	18 (8.8 to 32)	2.4 (−0.1 to 6.9) **
Apnoea	6%	4%	31%*
Duration of illness (days)	4 (2 to 5)	3 (2 to 4)	3 (2 to 4)
Atopic family history	70%	65%	64%
Smoking in home	71%	79%	55%

Data are median and quartiles unless stated otherwise (Pearson Χ^2^ two sided significance:

* = p<0.01,

** = p<0.001).

### Blood Urea

Blood urea was measured in 88 infants (no oxygen n = 6/25, oxygen dependent n = 32/107, &ventilated n = 50/53). Pooled data from these infants was normally distributed (mean = 2.9 mmol/l, 2.5 to 97.5 percentile = 1.0 to 5.8 mmol/l) and did not differ significantly from the normal range for healthy infants and young children aged 1 to 3 yr (n = 50, mean = 3.9 mmol/l, 2.5 to 97.5 percentile = 1.8 to 6.0 mmol/l).[25] There were no significant differences in blood urea between disease severity groups. The clinical decision to bleed only these children who may have been dehydrated might be expected to bias towards high urea values but the data does not support this. We conclude that that blood urea levels for this group of previously well infants admitted with bronchiolitis were normal and that none were in renal failure.

### Cytokine concentrations

NPA concentrations of IFN-γ, SP, and IL-9, in infants grouped by disease severity are described without adjustment for sample dilution in table 2. Data were not normally distributed. IFN-γ concentrations differed significantly between groups of infants who never required oxygen and those who required oxygen (Mann-Whitney U p = 0.05) and those who required ventilation (p = 0.04). SP concentrations differed significantly between groups of infants who never required oxygen and those who required ventilation (p = 0.04). There were no significant differences in IL-9 concentrations between groups.

**Table 2:** NPA concentrations of IFN-γ, SP, and IL-9 (unadjusted) by disease group.

	No oxygen	Oxygen	Ventilated
IFN-γ (pg/ml)	30 (64)	7.0 (44) *	1 (30) *
SP (pg/ml)	211 (307)	130 (421)	72 (323) *
IL-9 (pg/ml)	112 (1389)	210 (994)	113 (471)

Data are medians (interquartile range)). Mann-Whitney U test * = significant difference at p<0.05 between No oxygen group.

**Figure 1:**
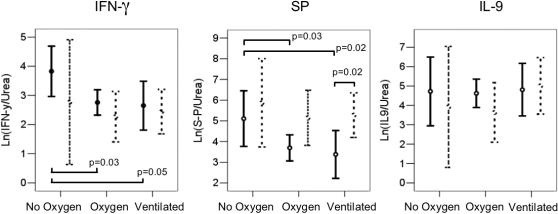
Nasopharyngeal concentrations of IFN-γ, SP, and IL-9 (expressed Ln([cytokine]/[urea]), ng/mol, geometric mean and 95% confidence intervals), in 185 infants grouped by severity of bronchiolitis and sub-grouped by term (solid lines) or preterm birth (dashed lines). Horizontal bars indicate between group comparisons by t-test and calculated p values.

Cytokine concentrations in each sample were adjusted by division by the sample urea concentration to reflect dilution during sample collection and handling. In grouped data this correction resulted in normal distributions, smaller coefficients of variation and a gain in the precision of the measurement, all of which permitted comparison of data in smaller groups of infants. Adjusted cytokine concentrations in term and preterm infants grouped by disease severity are shown in figure 1 (expressed natural log Ln([cytokine]/[urea]), ng/mol).

In term infants; significant differences (all p<0.05) were observed between geometric mean IFN-γ and SP concentrations in the no oxygen group (IFN-γ = 48 ng/mol, SP = 221 ng/mol) and both the oxygen dependent (IFN-γ = 16 ng/mol, SP = 42 ng/mol) and ventilated groups (IFN-γ = 14 ng/mol, SP = 29 ng/mol).

In preterm infants; no significant differences in SP and IFN-γ concentrations were observed between disease groups.

SP concentrations in ventilated term infants (29 ng/mol) were significantly lower than in ventilated preterm infants (231 ng/mol), p = 0.02.

IL-9 concentrations did not differ significantly with disease severity in term or preterm born infants.

**Figure 2:**
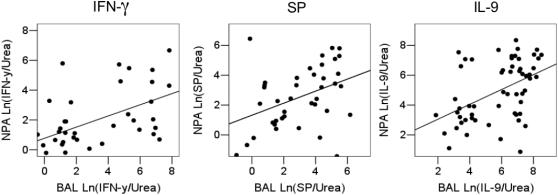
Scatter plot and best fit line between IFN-γ, SP and IL-9 concentrations (expressed Ln([cytokine]/[urea]), ng/mol) in paired samples of NPA and BAL. Pearson correlation coefficient: SP = 0.39 (n = 40, p = 0.01), IFN-γ = 0.35 (n = 38, p = 0.03)&IL-9 = 0.46 (n = 53, p = 0.001).

We compared the concentrations of each cytokine (Ln([cytokine]/[urea])) in paired NPA and BAL samples collected from ventilated infants (figure 2). There were significant positive linear correlations for each cytokine measured (Pearson correlation coefficient (n&p value)); SP = 0.39 (n = 40, p = 0.01), IFN-γ = 0.35 (n = 38, p = 0.03)&IL-9 = 0.46 (n = 53, p = 0.001) although there was moderate scatter.

### Viral load

**Figure 3:**
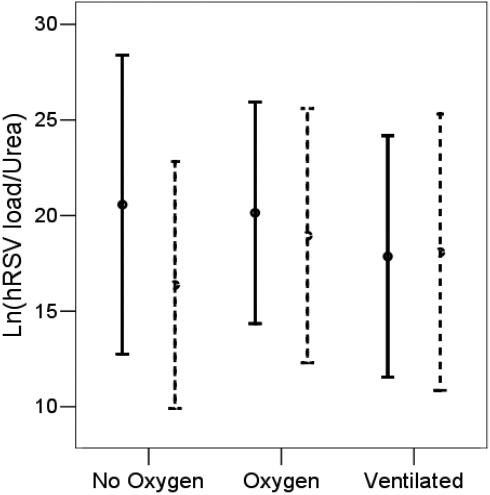
Cell-free hRSV load (expressed Ln(hRSV load/urea), geometric mean and 95% confidence intervals), in the nasopharyngeal aspirate of 104 infants grouped by worst severity of bronchiolitis during hospital admission and sub-grouped by term (solid lines) or preterm birth (dashed lines).

NPA hRSV load quantified in 104 infants ranged from 4.36E+4 to 2E+11 copies/µl with a mean of 5E+9 copies/µl. A geometric histogram of the data set was normally distributed. There were no significant differences in hRSV load between 104 infants grouped by disease severity or when sub-grouped by gestation at birth regardless of whether the data compared were or were not adjusted for dilution (figure 3).

**Figure 4:**
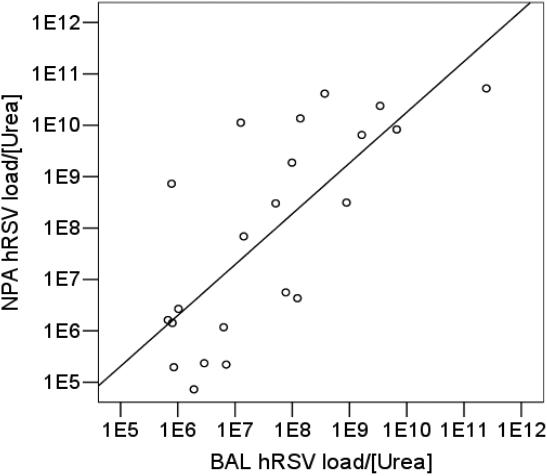
Scatter plot and best fit line between hRSV load (natural log hRSV load/[urea]) in 22 paired samples of NPA and BAL, Pearson correlation coefficient = 0.71 (R^2^ = 0.55, p<0.001).

The precision of the RT-PCR assay for samples measured in duplicate was excellent (R^2^ = 0.996, p<0.0001). There was a significant positive linear correlation between hRSV load in 22 paired samples of NPA and BAL (data compared natural log hRSV load/[urea], Pearson correlation coefficient = 0.71, R^2^ = 0.55, p<0.001)) (figure 4).

Comparison was made between NPA hRSV load and concentrations of the cytokines (SP, IFN-γ&IL-9) and in the same NPA sample. There was no association between NPA hRSV load and concentrations of SP (n = 99), IFN-γ (n = 100) or IL-9 (n = 95) in NPA.

### Analyses of independent effects

Multivariate binary logistical regression models predicting disease severity were constructed using patient characteristics, adjusted NPA cytokine concentrations and adjusted NPA hRSV load (table 3).

**Table 3:** Independent variables predicting severe hRSV Bronchiolitis (two definitions) in 185 participants by multivariate binary logistical regression.

Predictor Variable	Any need for supplemental oxygen, including ventilation.		Any need for mechanical ventilation.	
	Relative Odds Ratio (95%CI)	Significance (p)	Relative Odds Ratio (95%CI)	Significance (p)
**Admission Weight (kg)**	0.016 (0.001–0.274)	0.004	0.018 (0.001–0.383)	<0.001
**Gestation (weeks)**	0.482 (0.287–0.812)	0.006	0.537 (0.357–0.808)	0.003
**NPA IFN-γ (ng/mol)**	0.724 (0.568–0.923)	0.009	0.857 (R) (0.680–1.059)	0.19 NS
**NPA SP (ng/mol)**	0.744 (0.615–0.900)	0.002	0.861 (0.740–1.002)	0.054

Predictor Variables included in models but rejected (R) where the Relative Odds Ratio was not independent or significant (NS, P>0.1) included gender, birth-weight, corrected age on admission, family history of atopy, exposure to environmental tobacco smoke, and adjusted NPA concentrations of both IL-9 and hRSV load.

The Relative Odds Ratio (ROR) table gives the predicted change in odds that a unit change in each Predictor Variable has upon the outcome of the model (severe disease) relative to the other Predictor Variables. The ROR value is a measure of the size and direction of effect i.e. a value near 1 has little effect while a value less than one indicates that an increase in the predictor decreases the risk of severe disease. The ROR table permits comparison of the size of effect that independent clinical and immunological factors have upon risk of severe disease.

In the multivariate analysis low weight on admission was the strongest independent predictor (lowest ROR) of severe disease by both definitions followed by reduced gestation at birth. Low NPA concentrations of SP and IFN-γ were significant independent predictors of severe disease as defined by “need for supplemental oxygen” with a similar ROR. Low NPA concentrations of SP and IFN-γ were associated with “need for ventilation” but the ROR were closer to 1 and for IFN-γ was not statistically significant.

## Discussion

### Principal Findings

Severe disease (whether defined by need for oxygen or need for mechanical ventilation) was associated with reduced IFN-γ and SP in the airways. There was high viral load and high IL-9 in infants with severe disease but the measured amounts of both factors were not different from those in infants who never required supplemental oxygen. Further analysis of data with adjustment for sample dilution demonstrated that the reduction in IFN-γ and SP in infants with severe disease was only observed in term-born infants.

Nasal airway sampling appears to be a useful surrogate for distal airway sampling since concentrations of IFN-γ, SP, IL-9 and viral load in NPA correlate with the same in BAL.

Multivariate analysis identified (in order of size of effect) low weight on admission, low gestation at birth, low NPA IFN-γ and low NPA SP as being significant independent predictors of severe disease. Gender, family history of atopy, exposure to environmental tobacco smoke, NPA viral load and NPA IL-9 did not vary with disease severity on univariate or multivariate analysis.

### Strengths and limitations of this study

Particular strengths of this study include the following features. This is a pragmatic study of a large number of previously well human infants sampled during routine care. Exclusions were limited which exposed the study to clinical heterogeneity. While heterogeneity is recognised to make small differences between groups harder to detect, when differences are observed they are more likely to be real.[26] The findings of this study are therefore generally applicable to most infant populations in industrialised countries. Clinical details and respiratory samples were collected in a systematic way. Patients in different disease groups were sampled at the same times in the course of their disease. Most samples were collected at admission and all within 24 hours so that it unlikely that our observations are artefacts induced by hospital treatment or mechanical ventilation. Correcting for sample dilution using sample urea improved the precision of measured factors. The study was sufficiently powered to allow univariate comparisons between term and preterm born infants and a multivariate analysis of clinical and immunological factors.

Limitations of the study include the following points. For pragmatic and ethical reasons sampling was limited to those secretions which are readily available in the clinical setting. There is no healthy control group for this type of study as well infants are not coryzal and have insufficient nasal secretions for direct aspiration.[9] Our conclusions are limited to comparisons between hospitalised previously well infants grouped by severity of hRSV disease. Ethical reasons also prevented collection of a complete data set for blood urea values, so respiratory sample urea alone was used to correct for sample dilution.

### Relationship of this study to others

IFN-γ induces protective cellular responses against viral infections and inhibits the proliferation of smooth muscles cells, endothelial cells and the synthesis of collagens by myofibroblasts.[27], [28] Reduced interferon production has been proposed as a permissive mechanism for severe respiratory virus infection and asthma exacerbations in humans.[29] Bont et al described low nasopharyngeal IFN-γ concentrations in 17 ventilated infants compared with 43 non-ventilated infants but warned that over-representation of premature born infants in the ventilated group may have introduced bias.[9] Our data confirms Bont et al's important finding and clarifies that the low airway IFN-γ response is restricted to previously healthy term-born infants. The non-structural proteins of hRSV antagonise the human interferon system allowing subversion of one element of the early innate immune response to the advantage of the virus.[30] Any inherent normal variation of airway interferon response within a population of otherwise healthy term born infants would be expected to predispose a minority of infants to severe disease. This inherent variation could also explain the very strong association between severe hRSV disease in infancy and the association with recurrent wheezing in childhood which is invariably seen in conjunction with viral respiratory infections and is independent of atopic status.[4], [5], [31]

Reduced local interferon concentrations would be expected to permit increased viral replication, greater cytopathic effects and increased shedding into the respiratory tract. De Vincenzo *et al* described a relationship between hRSV load and disease severity using plaque assays and a multivariate analysis.[11] The relationship was not strong and like our data was not found on univariate analysis. In our study both forms of analysis rejected hRSV load on day of admission as predicting disease severity. Our finding agrees with three other studies which measured viral load by plaque assay and reverse transcribed RT-PCR though some of these studies may have been underpowered and all were restricted to univariate analysis.[11], [32]–[34] In the studies by Perkins et al (RT-PCR, 36 infants) and Wright et al (plaque assay, 17 infants), viral load in nasal washes decreased over the first few days of admission at a time when the clinical manifestations of hRSV disease were worsening.[33], [35]

Like Wright and Perkins we observed a strong linear relationship between hRSV load in the upper and lower airways of individuals across a range of six magnitudes with some scatter.[33], [35] This suggests that our method like theirs makes a valid measurement of total virus shed from a respiratory tract that behaves as a continuum. We chose not to use plaque assays in this study which recruited just under 200 infants over 3 winter epidemics. Plaque assays are laborious and need fresh samples. Co-circulating hRSV strains vary year on year and have very large differences in tropism in plaque assays.[35]

We do not dismiss the likelihood that viral load at an earlier time point is a predictor of disease severity, or that viral load would be a predictor of disease severity if comparison were made between some of the great majority of infants who are not hospitalised (97.5%) and the minority that are.

To our knowledge the role of endogenous SP has not been investigated in the airways of human infants. Massive neutrophil migration into the airways is a characteristic feature in severe hRSV bronchiolitis.[6], [36] “Neurogenic inflammation” mediated by the neurokinin SP is one mechanism proposed for neutrophil recruitment and smooth muscle contraction in the airways of hRSV infected weanling rats.[8], [37] Exogenous SP has potent pro-inflammatory cytokine effects in mouse and human airway studies.[38], [39] The finding of lower concentrations of SP in the cell free airway fluid of term infants with severe disease is therefore surprising. However it is congruent with the reports of up-regulation of the high affinity SP receptor in lung tissue from RSV infected weanling rats by Piedimonte and King.[8], [37] The finding also compliments Piedimonte's more recent description of reduced neurotrophin in the cell free supernatant of human infant BAL and increased expression of the neurotrophin receptor in the cellular fraction of human infant BAL.[40] Blockade of the SP receptor in hRSV infected weanling rats reduces apnoea.[41], [42] hRSV infection in human infants is associated with apnoea and sudden infant death. Our data supports the hypothesis that SP plays a role in the mechanism of hRSV disease.

McNamara et al described large quantities of IL-9 in the lower airways of infants with severe bronchiolitis and discovered that neutrophils were the source.[10] IL-9 induces mucous cell metaplasia, production of mucus, proinflammatory chemokines and airway hyper-responsiveness.[43]–[45] Genetic linkage studies in humans indicate that IL-9 plays an important role in the pathogenesis of atopic asthma.[46] In earlier studies we were unable to determine if airway concentrations of IL-9 varied with hRSV disease severity because we only examined the bronchoalveolar fluid from ventilated infants. We found large amounts of IL-9 in both the upper and lower airway secretions but did not observe any association with disease severity.

McNamara et al reported striking differences in the cellular and pro-inflammatory and anti-inflammatory responses in BAL between term and prematurely born infants ventilated for severe hRSV bronchiolitis.[6], [12] Premature birth affects the functional development of the lung resulting in reduced alveolar formation, impaired gas mixing and low lung volumes.[47] Preterm infants would be expected to require less airway obstruction than term infants to reach the compromised physiological state that requires oxygen or ventilation. Severe bronchiolitis in previously healthy preterm infants need not involve any relative difference in immunity but instead simply reflect a degree of reduced lung function.[48] This is evident from the multivariate analysis which found strong independent associations between low weight on admission and low gestation with severe disease. Both these predictor variables are markers of lung growth and function. In comparison, term infants with appropriately developed airways are more physiologically robust. In term infants, differences in the innate immune system (reduced IFN-γ synthesis and possibly increased SP uptake) differentiate those infants who experience severe disease from those who are less affected.

### Meaning of this study

Our data directly supports two of the recently proposed mechanisms for severe hRSV disease, namely a reduced local IFN-γ response and SP mediated inflammation. We have found large amounts of hRSV and IL-9 in airways secretions from the upper and lower respiratory tract but could not associate absolute amounts with disease severity.

A difference in the innate response to primary hRSV infection, common to term infants susceptible to severe hRSV bronchiolitis, may explain why so many of these infants experience recurrent wheeze in childhood in association with viral respiratory tract infections.

## Supporting Information

Appendix S1 Detailed Methods and Structured History Questionaire.
(0.03 MB DOC)
