# Supplementary material for: Severe Respiratory Syncytial Virus Bronchiolitis in Infants Is Associated with Reduced Airway Interferon Gamma and Substance P
Source: PLoS One. 2007 Oct 17;2(10):e1038. doi: 10.1371/journal.pone.0001038 (PMC2001182; doi:10.1371/journal.pone.0001038)
Supplement: Appendix S1 — Detailed Methods and Structured History Questionaire. (0.03 MB DOC) [file pone.0001038.s001.doc]

## Appendix

### Measurement of cell free hRSV load

Nucleic acid was extracted from 140 µL of filtered cell-free respiratory sample using QIAamp*™* viral RNA MiniKit*™* (Quiagen Ltd, UK). 10 µL of purified nucleic acid solution was subject to reverse transcription using a hRSV specific primer as previously described.[21] 1 µL of cDNA solution was subject to real time polymerase chain reaction (PCR) using primers specific to a highly conserved region of the hRSV N gene using an Opticon 2*™* thermal cycler (Genetic Research Instrumentation Ltd., UK). Each PCR reaction consisted of 20 mM Tris-HCl, 50 mM KCl, 2.5 mM MgCl_2_, 1 mM of each primer (forward primer TGGGTGGTGAAGCAGGATTCTA, reverse primer (CAGCATTGCCTAATACTACACT), 250 pM dNTPs, 0.15% Triton X-100, 20 pg BSA, 1 units platinum Taq Polymerase (Cat No. 10966-026, Invitrogen), 0.5 x SYBR Green I (Cambridge Bioscience, UK), in a final reaction volume of 20 µL.

PCR conditions consisted of an initial denaturing step at 95^o^C for 10 minutes, followed by 35 cycles of denaturation at 94^o^C for 10 seconds, annealing at 54^o^C for 20 seconds, polymerization at 72^o^C for 15 seconds then 75^o^C for 1 second, followed by fluorescence reading. Absolute quantification was based on gene-specific standard curves, constructed by amplification of a serially diluted synthetic 121 base oligonucleotide (MWG-biotech) consensus sequence. To ensure specificity of amplifications, all PCR reactions were subjected to melting curve analysis from 65 to 95^o^C, with increments of 0.2^o^C per second. Samples were accepted for quantitation only if the melting curve conformed to the melting curve generated by the quantitation standard which is identical to those derived from hRSV culture positive samples and a plasmid DNA containing the target hRSV N gene. hRSV load is expressed as copies of N gene molecule per microlitre of sample (copies/µl).

**Structured History Form**

AHCH Unit Number: Ward:

Name: Date of Birth:

Date of Admission: Weight on Admission (Kg):

**History**

Birth:

Birth weight: __ lb __oz Gestation: ___/40

Admitted to SCBU Yes/No , if so Days of Oxygen ____, Ventilated: Yes/No

Past Medical History:

Underlying Conditions: Yes/No
(e.g. CLD if in oxygen for 28 days by 36 weeks corrected gestational)

Atopic History in any first degree relative: Yes/No

ASK “Do you or your partner or any of the babies brothers or sisters have asthma, hayfever or eczema GP diagnosed on treatment?”

Smoking in any Household member regardless where: Yes/No

Clinical History:

ASK “Before this admission how many days was your baby unwell for?”___
Prompt about cough or snuffles if parent unsure about what unwell means

ASK “Did you baby ever stop breathing?” (describe apnoea if unsure) Yes/No
